# Accuracy of transdermal alcohol monitoring devices in a laboratory setting

**DOI:** 10.1093/alcalc/agad068

**Published:** 2023-10-23

**Authors:** Eileen Brobbin, Paolo Deluca, Simon Coulton, Colin Drummond

**Affiliations:** Addiction Department, Institute of Psychiatry, Psychology & Neuroscience, King’s College London, 4 Windsor Walk, London SE5 8AF, United Kingdom; Addiction Department, Institute of Psychiatry, Psychology & Neuroscience, King’s College London, 4 Windsor Walk, London SE5 8AF, United Kingdom; Centre for Health Service Studies, University of Kent, Canterbury CT2 7NF, United Kingdom; Addiction Department, Institute of Psychiatry, Psychology & Neuroscience, King’s College London, 4 Windsor Walk, London SE5 8AF, United Kingdom

**Keywords:** alcohol, alcohol monitoring, alcohol treatment, transdermal alcohol sensor, wearable alcohol biosensor

## Abstract

The development of transdermal alcohol sensors (TASs) presents a new method to monitor alcohol consumption with the ability to objectively measure data 24/7. We aimed to evaluate the accuracy of two TASs (BACtrack Skyn and Smart Start BARE) in a laboratory setting. Thirty-two adults received a dose of ethanol 0.56 g/kg body weight as a 20% solution while wearing the two TASs and provided Breath Alcohol Concentration (BrAC) measurements for 3.5 h postalcohol consumption. Pearson’s correlations and repeated measures analysis of variance tests were conducted on the peak, time-to-peak, and area under the curve data. Bland–Altman plots were derived. A time series analysis and cross-correlations were conducted to adjust for time lag. Both TASs were able to detect alcohol and increase within 20 min. BrAC peaked significantly quicker than Skyn and BARE. BrAC and Skyn peaks were negatively significantly correlated (*r* = −0.381, *P* = .035, *n* = 31), while Skyn and BARE peaks were positively significantly correlated (*r* = 0.380, *P* = .038, *n* = 30). Repeated measures analysis of variance found a significant difference between BrAC, Skyn, and BARE (*F*(1.946, 852.301) = 459.873, *P* < .001)). A time series analysis found when BrAC-Skyn and BrAC-BARE were adjusted for the delay to peak, and there was still a significant difference. Failure rates: 1.7% (Skyn) and 4.8% (BARE). Some evidence was obtained for TAS validity as both consistently detected alcohol. Failure rates and time lag show improvements in older device generations. However, neither TAS presented strong equivalence to the breathalyser even when the lag time was adjusted. With further testing and technology advancements, TAS could be a potential alcohol monitoring tool. Two of the newest TAS devices were worn in laboratory conditions for one afternoon to compare their accuracy of alcohol monitoring to a breathalyser. Findings suggest that the two TASs (BACtrack Skyn and SmartStart BARE) recorded significantly similar data postalcohol consumption, but not with the breathalyser.

## Introduction

Transdermal' alcohol sensor (TAS) devices can be worn to measure transdermal alcohol concentration (TAC) excreted through a sensor against the skin. This sensor analyses the concentration of alcohol in the skin vapours emitted and calculates the TAC (Dumett et al. 2008). Current methods used to measure alcohol consumption within clinical settings include breathalysers, blood tests, or self-report measures, such as the Alcohol Use Disorder Identification Test-Consumption (Saunders et al. 1993) or Timeline Follow Back (Sobell and Sobell 1992; Kuntsche et al. 2022). However, all these methods have limitations in the accuracy, amount of detail collected, or period over which they can collect data. They can also be costly and burdensome to use (Kuntsche et al. 2022). TASs could address some of these limitations, as they can be worn over extended periods while providing continuous measurement (Litten et al. 2010; Leffingwell et al. 2013). This means TAS could capture data when self-report is not possible (for example, when heavy drinking leads to lack of capacity and blackouts) or when it is not possible for multiple daily breathalysers, blood, or urine tests (Kuntsche et al. 2022). A breathalyser can only capture alcohol consumption which has occurred within a short window before breathalyser administration (Kuntsche et al. 2022). However, as this technology is still relatively new, TAS brands’ validity and reliability still require investigation. There are studies published on TAS accuracy or acceptability (Caluzzi et al. 2019; Fairbairn and Kang 2019; Croff et al. 2020; van Egmond et al. 2020; Courtney et al. 2022; Brobbin et al. 2022a, 2022b), but most of these are limited by only testing one brand of TAS and each study uses a slightly different design, population, or analysis, making it difficult to draw direct comparisons.

There are various TAS brands available, and all are slightly different in their technology, appearance, wear, or output reporting. SCRAM is the most validated TAS (Sakai et al. 2006; Dougherty et al. 2012; Barnett et al. 2014, 2017; Hill-Kapturczak et al. 2014; Alessi et al. 2019; Fairbairn and Kang 2019; Roache et al. 2019; van Egmond et al. 2020; Wang et al. 2021; Ash et al. 2022) and it has been available in the US criminal justice system for the past 20 years and in the UK (United Kingdom) justice system since 2020. SCRAM has various TAS models, but it is usually worn on the ankle and is the largest device in size. SCRAM uses a home modem to send data to the server and monitors alcohol consumption every 30 min. The ION Wearable TAS is worn on the wrist and is available for purchase on their website (‘ION Wearable’, no date). They use noninvasive enzymatic alcohol sensors with replaceable cartridges. BACtrack Skyn is the latest developed, available, TAS using fuel-cell technology and is available for research purposes and beta testing. It is worn on the wrist and can sync with Apple iOS devices to provide data on an app and a website server. If the Skyn is being worn close to the paired Apple device, real-time syncing and viewing of alcohol consumption is possible. The latest version of the Skyn App also has the option to estimate a range of blood alcohol concentration (BAC) based on the TAC data. However, this data conversion is currently only usable in personal Skyn use and not in a research context. WrisTAS was another wrist-worn TAS that has been widely validated (Swift et al. 1992; Webster and Gabler 2008; Marques and McKnight 2009; Bond et al. 2014; Luczak et al. 2015; Simons et al. 2015; Croff et al. 2020) but has since been bought by Smart Start. Other TAS are in various stages of development as evidenced in previous literature reviews (van Egmond et al. 2020; Brobbin et al. 2022a, 2022b). The BARE is a newer prototype developed by Smart Start and is not currently available to purchase. The BARE manufacturers state the target audience is clinical use, unlike the other brands which are targeted for general consumer use. More details about the two TASs used in this study (BACtrack Skyn and Smart Start BARE) are included in Supplementary Information.

When considering implementing TAS within clinical settings, their use would not only be detecting alcohol presence. Recording when, the amount, peaks of use and the accuracy are important. They could be used to discern between low, moderate, or high levels of drinking (Dougherty et al. 2012; Hill-Kapturczak et al. 2014). TAS could become a valuable tool for alcohol treatment to allow for continuous alcohol monitoring TAS brands, with the option to view output in near-real time, could provide alcohol services with the opportunity to initiate timely and personalized interventions for patients going through alcohol treatment, and afterwards, when there is a high chance of relapse (Moos and Moos 2006). TAS could also benefit alcohol research, helping researchers continuously measure alcohol consumption and potentially implement contingency management (CM) for alcohol reduction in near-real time (Barnett et al. 2011, 2017; Dougherty et al. 2014, 2015; Averill et al. 2018; Richards et al. 2023). While only the SCRAM can be locked and considered tamper-proof, the other brands have a temperature sensor to monitor removals but are not as secure as SCRAM. The use of a removable device to deliver CM will need further exploration and potential penalties for dealing with device removals, data loss, and wearers possibly turning the device off to obscure data collection.

Earlier studies have shown a general agreement between TAC and Breath Alcohol Concentration (BrAC) or BAC values using SCRAM (Dougherty et al. 2012; Barnett et al. 2014; Hill-Kapturczak et al. 2014; Fairbairn and Kang 2019) and Skyn (Fairbairn and Kang 2019; Wang et al. 2019, 2021; Fairbairn et al. 2020; Ash et al. 2022). However, other studies have shown varying levels of disagreement (Sakai et al. 2006; Marques and McKnight 2009; Croff et al. 2020). A large majority of these studies have been conducted using SCRAM or WrisTAS (Leffingwell et al. 2013; Greenfield et al. 2014; Barnett 2015; Dougherty et al. 2015; Fairbairn et al. 2019; Wang et al. 2019; van Egmond et al. 2020). There has been a smaller number of accuracy studies using Skyn (Fairbairn and Kang 2019; Wang et al. 2019, 2021; Fairbairn et al. 2020; Rosenberg et al. 2021; Ash et al. 2022; Richards et al. 2022). The few studies conducted using Skyn found an overall high correlation between self-report, BrAC and Skyn TAC that Skyn could distinguish between low and high alcohol doses and was considered to be acceptable and feasible to wear (Fairbairn and Kang 2019; Fairbairn et al. 2020; Rosenberg et al. 2021; Wang et al. 2021; Ash et al. 2022; Courtney et al. 2022; Richards et al. 2022).

This study adds to this research work with the latest version of Skyn compared to a newer wrist sensor (BARE). This study aims to investigate the accuracy of these two TASs to measure alcohol concentration within a laboratory setting for a population of alcohol users not meeting the criteria of alcohol dependence (AUDIT < 16). It will also be the first to test the accuracy of BARE. As all three devices (breathalyser, Skyn, and BARE) aim to measure alcohol consumption, we expect values from all three devices to be correlated. We predict that the Skyn and BARE will be correlated in terms of both peak data and time-to-peak readings.

## Materials and Methods

### Recruitment

Participants were recruited through a fortnightly research email at King’s College London, which included the study advertisement. Those interested were asked to email the researcher. To be eligible, participants had to meet the inclusion criteria: (i) 18 years old and over, (ii) regular drinker (five or more standard UK units for men and four or more standard UK units for women, consumed at least twice per month), (iii) no desire to reduce their drinking, (iv) AUDIT score < 16, and (v) willing to provide informed consent to participate in the study. Potential participants were excluded if they had: (i) drug or alcohol-related health problems, (ii) a health diagnosis potentially complicated by alcohol ingestion, (iii) illicit drug use (excluding cannabis) within the last 4 weeks, (iv) a history of drug or alcohol treatment, (v) were prescribed contraindicated medication, (vi) pregnant, and (vii) presence of COVID-19 symptoms.

These inclusion and exclusion criteria were used to target individuals who regularly consumed the amount of alcohol that was to be provided to them as part of the study. We did not want to provide alcohol to those who did not regularly consume this amount, had a higher risk of alcohol dependence by scoring >16 on the AUDIT, or had any health problems complicated by alcohol consumption.

### Alcohol measures

AUDIT: Used to ensure participants scored below the indication of harmful alcohol use or dependence and moderate–severe alcohol use disorder (Saunders et al. 1993).

Breathalyser (Lion Alcometer SD-400): Lion fuel cell sensor (standard version) measure range: 0.02–2.00 mg/l BrAC, operating range: −5 to +45°C, with optimum operation between +10 and +40°C. Calibrated one week before recruitment started.

TAS [BACtrack Skyn (model T15/2021) and Smart Start BARE (prototype)]: Skyn provided data output every 20 s and included motion and temperature (Celsius) sensors. Skyn output can be displayed in 20-s, 1-min, 5-min, or 30-min intervals. We viewed this data at 1-min intervals to best match the breathalyser and BARE readings. BARE only provided data output every 2 min (average of the data recorded every 20 s) and recorded temperature (°C) data. For the analysis, we used these two variables (alcohol signal mg/dl) and temperature, but BARE also had columns for sample count, Prox status, Prox (light), and descriptive alcohol data [event peak, peak level, data slope, and area under the curve (AUC)].

### Procedure

Participants completed an AUDIT and the Nuffield Medical health questionnaire to ensure they met inclusion criteria and were asked not to consume alcohol for 24 h prior and to eat a light lunch 1 h before arrival. All procedures took place in the afternoon. On arrival, a breathalyser measurement was taken to confirm a reading of zero. Participants were weighed and their height was measured. The participant’s weight determined the alcohol dose. All participants received 0.56 g/kg of 37.5% ABV vodka as a 20% solution mixed with orange fruit juice, with this drink being consumed within 10 min. For example, a participant weighing 80 kg would have a dose of 44.8 g ethanol (5.5 UK units).

Automatic measurements were recorded by the TAS. Breathalyser measurements were taken every 15 min, starting after alcohol consumption for a total of 3.5 h; therefore, there were 15 measurements taken. Participants were thanked for their time and provided with a snack and a £10 voucher. All participants were told that they would have to remain until their BrAC was below a safe level of 0.25 mg/l, below the UK drink drive limit (0.35 mg/l), even if this was longer than the set 3.5 h. Due to the amount of alcohol being consumed by each participant, it was not feasible for participants to stay until their BrAC values had reduced to zero. This study was approved by the King’s College London ethics committee (reference: HR/DP-20/21-24414).

### Data manipulation

Missing values were calculated using linear interpolation in Excel and any negative values were left-censored to zero, as conducted in previous research (Courtney et al. 2022). We standardized the values by converting all device values to the same unit, mg/l. Breathalyser values were in mg/l units. The BARE values were converted from mg/dl to mg/l by multiplying the numerator by 10, and the Skyn values were converted from μg/l to mg/l by dividing by 1000. As the Skyn provided values for each minute we were able to use the minute that matched with the breathalyser. The BARE provided values every 2 min and so when the value did not match exactly with the same minute as the breathalyser the minute before was used for analysis.

### Data analysis

A statistical power calculation was used to determine the sample size, which were 25 participants (alpha of 0.05, power of 0.90, and estimated effect size of 0.60). Sociodemographic and descriptive device data were recorded and plotted. All device values were standardized. We reported on the failure rate for all devices and the peak, time-to-peak, and AUC for each participant.

To test if the two TASs’ peak and time-to-peak data are correlated. Data were analysed at each time point (15-min intervals) from alcohol consumption, for a total of 3.5 h (15 timepoints). Pearson’s correlations were used for peak data and device timepoints, and paired *t*-tests were used for time-to-peak. We define the peak data as the highest value recorded for each participant by each device. That means, for the same participant the three devices (BrAC, Skyn, and BARE) could have time of peak data at different times.

After a Shapiro–Wilks test of normality, repeated measures analysis of variance (ANOVA) tests were conducted to compare the Breathalyser and Skyn, Breathalyser and BARE, and Skyn and BARE. Mauchly’s Test of Sphericity was conducted.

The AUC was calculated for each device from the proportion of cells within a matrix below the curve. The AUC was calculated to investigate if there was a difference in the AUC for each device. Pearson’s correlations for AUC and alcohol administered were conducted and correlation AUC between BrAC, Skyn, and BARE. Bland–Altman (BA) plots (Bland and Altman 1986) were used to compare devices for time-to-peak and peak alcohol concentration if possible.

A time series analysis and cross-correlation were conducted to adjust timepoints for any time lag in measurement between the devices. The timepoints were then adjusted for the found lag. The Shapiro–Wilks test of normality, repeated measures ANOVA, and Mauchly’s test of Sphericity were conducted with these adjusted data. The AUC was recalculated for this adjusted data. Pearson’s correlations with AUC were conducted with this time-adjusted dataset. To note, the AUC is incomplete as participant measurements were not taken until BrAC and TAS values returned to zero. All the analysis was conducted with IBM SPSS Statistics version 28.

**Table 1:** Sample characteristics.

Participant characteristics Mean (SD)	All (*n* = 32)	Males (*n* = 15)	Females (*n* = 17)	Sex difference
Age in years	25.84 (6.14)	26.53 (7.53) Range: 18–47	25.24 (4.76) Range: 18–39	*P* = .304
BMI	22.23 (3.31)	24.14 (3.28) Range: 20–31.5	20.55 (2.33) Range: 14.9–24.3	*P* = .330
Height in centimetres	172 (7.92)	177.32 (6.66) Range: 167.5–187.0	167.62 (6.03) Range: 155–175	*P* = .478
Weight in kilograms	66.28 (12.53)	75.56 (8.96) Range: 59.3–94.4	58.09 (9.05) Range: 40.7–78.0	*P* = .823
AUDIT scores	6.53 (3.12)	7.27 (3.33) Range: 2–12	5.88 (2.87) Range: 3–15	*P* = .681
Race/ethnicity *n* (%) Caucasian Asian Black Hispanic	23 (72%) 6 (19%) 2 (6%) 1 (3%)	12 (79%) 1 (7%) 1 (7%) 1 (7%)	11 (65%) 5 (29%) 1 (6%) 0	*X* ^2^(3) = 3.599, *P* = .308
Alcohol data Mean (SD)	All (*n* = 32)	Males (*n* = 15)	Females (*n* = 17)	Sex difference
Standardized data				
Peak BrAC mg/l	0.40 (0.15)	0.37 (0.11)	0.43 (0.18)	*P* = .162
Peak Skyn mg/l	0.10 (0.04)	0.10 (0.04)	0.10 (0.03)	*P* = .489
Peak BARE alcohol signal mg/l	799.35 (608.02)	570.00 (386.42)	908.24 (748.18)	*P* = .925
BrAC time-to-peak (min)	53.44 (32.98)	51.00 (29.89)	55.59 (36.27)	*P* = .417
Skyn time-to-peak (min)	120.32 (39.93)	125.67 (38.25)	115.31 (42.05)	*P* = .618
BARE time-to-peak (min)	140.97 (45.65)	139.14 (40.19)	142.47 (50.88)	*P* = .638

AUDIT, Alcohol Use Disorder Identification Test. Significant *P* < .05.

**Figure 1:**
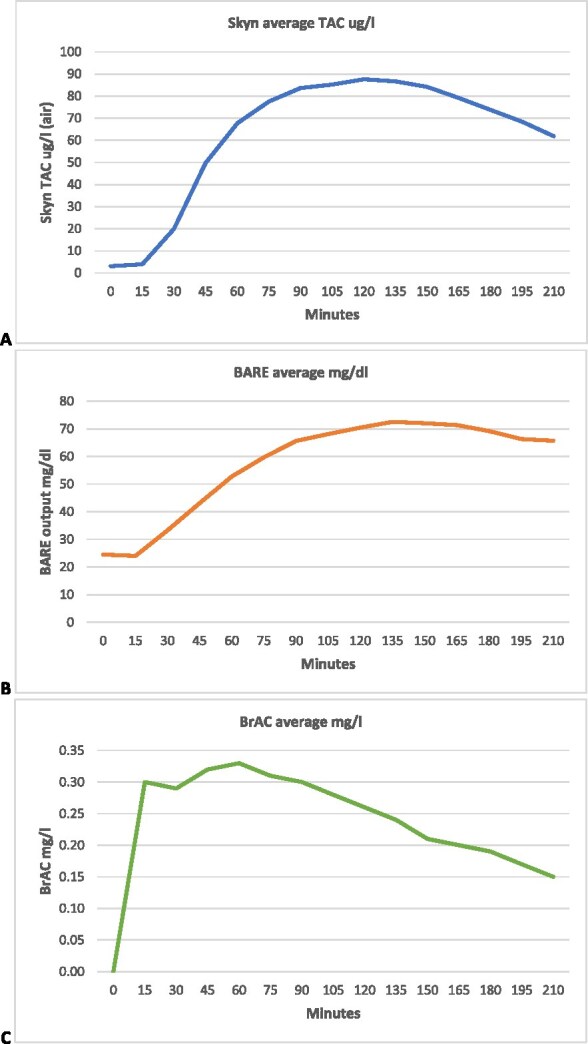
Average Skyn, BARE, and BrAC data for all participants; (A) average Skyn output every 15 min for 3.5 h (μg/l air); (B) BARE output every 15 min for 3.5 h (mg/dl); (C) average BrAC measured every 15 min for 3.5 h (mg/l).

## Results

### Baseline and descriptive device statistics

There was a total of 32 healthy adult participants (15 males, 17 females). Sample characteristics and descriptive standardized device data are reported in Table 1. Data recorded from each participant included their BrAC breathalyser, Skyn, and BARE data (mg/l). The average peak BrAC was 0.40 mg/l (SD: 0.15 mg/l) (0.083 g/210 l), the average peak Skyn was 0.10 mg/l (SD: 0.04 mg/l and the average peak BARE was 8.01 mg/l (SD: 6.08 mg/l).

Below, we have provided a visualization of the average data from the 3.5 h (Fig. 1).

### Failure rate

In this context, failure rate means missing data, not reported in device output. The Skyn output was reported in 1-min intervals, so the failure rate is the number of 1-min intervals that were not reported. Skyn failure rate was 1.7% for all timepoint measures (for 31 participants, 108 out of 6466 timepoints were missing and not reported in Skyn output). One participant (P27) failed to record usable data due to human error; we excluded this participant from this calculation. The BARE output was reported in 2-min intervals, so the failure rate is the number of 2-min intervals that were not reported. The BARE failed to record one participant (P30) for an unknown reason, possibly a device error. Including this participant, BARE had a failure rate of 4.8% (for 32 participants, 161 out of 3357 timepoints were missing and not reported in BARE output). Both TASs recorded a relatively stable baseline TAC at the start of the experiment, with a clear rise following alcohol consumption, a peak, and the start of a fall. All breathalyser measurements were successfully recorded.

### Breath Alcohol Concentration and transdermal alcohol concentration data

The amount of ethanol (grams) each participant consumed, calculated by their weight, the peak, time-to-peak, and the AUC from each device are included in Table 2.

The data were analysed at 15-min intervals to match breathalyser measurements, referred to as timepoints. There were a total of 15 timepoints over the 3.5-h study length: 0 min (postalcohol consumption) = timepoint 1, 15 min = timepoint 2, 30 min = timepoint 3, 45 min = timepoint 4, 60 min = timepoint 5, 75 min = timepoint 6, 90 min = timepoint 7, 105 min = timepoint 8, 120 min = timepoint 9, 135 min = timepoint 10, 150 min = timepoint 11, 165 min = timepoint 12, 180 min = timepoint 13, 195 min = timepoint 14, 210 (3.5 h) = timepoint 15.

Pearson’s correlations were conducted between BrAC and Skyn, BrAC and BARE, and Skyn and BARE, as reported in Table 3. Only one significant positive correlation was found between BrAC and Skyn timepoints, at timepoint 2, 15 min post alcohol consumption. No significant Pearson’s correlations were found between BrAC and BARE. Pearson’s correlations between Skyn and BARE were conducted with significant positive correlations found at timepoints 5, 6, 10, 11, 12, and 13 (min: 60, 75, 135, 150, 165, 180). Pearson’s correlations found peak BrAC and peak Skyn were negatively correlated, while peak Skyn and peak BARE were positively correlated.

The TAS appeared to detect each alcohol event for each participant. TAC output data increased within 20 min of alcohol consumption. The average time-to-peak value for Skyn was 120.32 min (SD = 39.93) and for BARE it was 140.97 min (SD = 45.64), compared to the breathalyser at 53.44 min (SD = 32.98) (Skyn lagged by 66.88 min and BARE by 87.53 min). The difference between Skyn and BARE time-to-peak was nonsignificant (*t*(29) = −1.723, *P* = .096). The time-to-peak between BrAC and Skyn and BrAC and BARE were significantly different (*t*(30) = −7.801, *P* < .001, *t*(30) = −10.202, *P* < .001, respectively). BARE peak was significantly correlated to body mass index (BMI) (*r* = 0.615, *P* < .001, *n* = 31).

Shapiro–Wilks test found that data output was normally distributed for BrAC, Skyn, and BARE. A repeated measures ANOVA was conducted. Mauchly’s Test of Sphericity indicated that the assumption of sphericity had been violated, and therefore, a Greenhouse Geisser correction was used. The difference between the devices was statistically significant (Table 4).

### Area under the curve

The AUC was calculated for each participant for BrAC, Skyn, and BARE data. The AUC was calculated using the start of the drinking event to the endpoint of the last measurement 3.5 h later. Pearson’s correlations of AUC and the amount of alcohol administered (in grams) were conducted. Skyn AUC and BARE AUC were correlated (*r* = 0.439, *P* = .015, *n* = 30), BrAC AUC was not significantly correlated with Skyn AUC (*r* = −0.189, *P* = .317, *n* = 30) or BARE AUC (*r* = −0.139, *P* = .480, *n* = 30).

Analysing AUC up to the peak, there were no significant correlations between any of the devices.

### Bland–Altman plot

**Figure 2:**
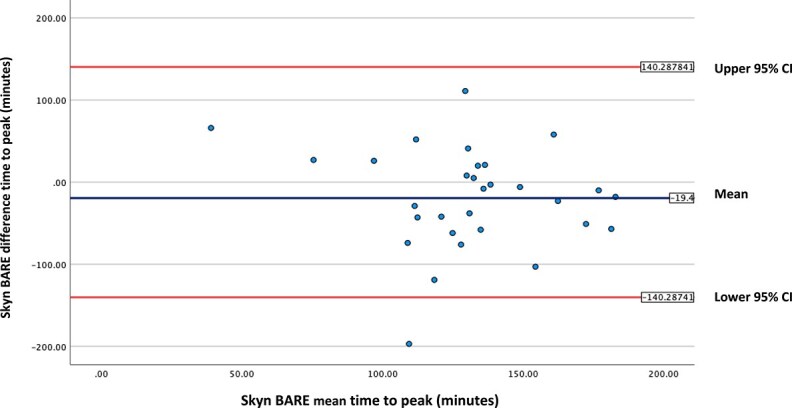
Bland–Altman plot for time-to-peak Skyn and BARE; BA plot for time-to-peak (min) Skyn and BARE devices; plot of differences between Skyn and BARE (*y*-axis) versus the mean of the Skyn and BARE (*x*-axis); the middle horizontal line represents the mean difference between the TAS (labelled Mean); the top horizontal line represents the upper 95% CI and the bottom horizontal line represents the lower 95% CI.

We created a BA plot (Bland and Altman 1986) for the time-to-peak for Skyn and BARE (Fig. 2) to visually compare the TAS. For each participant, we averaged the time-to-peak for Skyn and BARE results (*x*-axis) and calculated the difference between the time-to-peak for Skyn and BARE results (*y*-axis). This BA plot provides a visual representation of the agreement between Skyn and BARE for the time-to-peak data. The mean is −19.400, the upper 95% CI is 101.488 and the lower 95% CI is −140.288.

If the two TASs were in perfect agreement, we would expect the blue circles to be close to the middle horizontal line with an intercept of zero. The BA plot below (Fig. 2) shows varying disagreement between the time-to-peak for Skyn and the time-to-peak for BARE.

### Time series analysis

We completed a time series analysis and cross-correlation to determine if there was a time lag for the TAS. We found that, while Skyn and BARE data were in sync, there was a lag of two timepoints (30 min) for Skyn behind BrAC and a lag of four timepoints (60 min) for BARE behind BrAC. Therefore, we adjusted these data by these respective timepoints to account for the time lag. We then conducted the repeated measures ANOVA and AUC with these adjusted data.

**Table 2:** Participant information: ethanol consumed, weight, device peak, time-to-peak data, and AUC.

				Peak			Time-to-peak (in min)			AUC		
Participant ID	Alcohol consumed	Weight	BrAC g/210 L	BrAC	Skyn	BARE	BrAC	Skyn	BARE	BrAC	Skyn	BARE
**1**	39.70	70.90	0.081	0.39	0.16	830.00	15	112	150	3.56	1643.59	8110.00
**2**	34.27	61.20	0.056	0.27	0.12	500.00	45	146	152	2.55	1271.70	5285.00
**3**	49.62	88.60	0.077	0.37	0.12	3500.00	45	138	86	3.10	1353.01	35595.00
**4**	38.70	69.10	0.075	0.36	0.07	260.00	15	110	84	2.61	719.15	2790.00
**5**	33.21	59.30	0.113	0.54	0.05	870.00	15	190	132	3.03	338.12	10295.00
**6**	36.23	64.70	0.100	0.48	0.10	770.00	75	89	62	4.62	1030.39	8080.00
**7**	29.29	52.30	0.113	0.54	0.11	630.00	15	151	110	4.42	1112.55	6760.00
**8**	44.74	79.90	0.083	0.40	0.16	1870.00	60	185	74	3.72	1527.90	17665.00
**9**	43.68	78.00	0.079	0.38	0.12	730.00	60	132	140	2.56	630.58	3660.00
**10**	37.46	66.90	0.067	0.32	0.10	450.00	75	100	142	2.82	763.35	4005.00
**11**	34.66	61.90	0.081	0.39	0.10	290.00	90	147	126	3.75	912.21	3055.00
**12**	42.06	75.10	0.067	0.32	0.07	1140.00	15	135	130	2.47	546.38	10360.00
**13**	42.73	76.30	0.060	0.29	0.17	1690.00	45	97	126	2.91	1784.28	18285.00
**14**	33.32	59.50	0.060	0.29	0.12	840.00	135	147	198	2.88	1209.64	7480.00
**15**	28.00	50.00	0.092	0.44	0.09	550.00	30	134	126	3.44	863.14	5950.00
**16**	30.97	55.30	0.054	0.26	0.12	700.00	75	72	146	2.64	1029.61	6865.00
**17**	43.68	78.00	0.067	0.32	0.14	780.00	45	59	178	3.17	1326.60	7040.00
**18**	29.74	53.10	0.077	0.37	0.13	690.00	15	90	166	3.29	1194.46	7210.00
**19**	29.12	52.00	0.054	0.26	0.13	430.00	45	91	134	2.43	1181.52	4110.00
**20**	37.24	67.10	0.133	0.64	0.05	520.00	45	94	156	4.96	321.44	6285.00
**21**	31.92	54.70	0.196	0.94	0.03	500.00	15	11	208	5.12	247.77	4830.00
**22**	46.87	83.70	0.079	0.38	0.09	800.00	60	144	124	3.84	684.44	9510.00
**23**	37.24	66.50	0.092	0.44	0.08	530.00	60	172	182	4.55	558.56	4650.00
**24**	31.92	57.00	0.094	0.45	0.07	790.00	45	103	206	4.11	576.67	6605.00
**25**	26.43	47.20	0.138	0.66	0.11	520.00	30	72	6	5.49	985.37	5690.00
**26**	22.79	40.70	0.054	0.26	0.08	590.00	75	137	140	2.97	781.59	6290.00
**27**	39.20	70.00	0.111	0.53		750.00	15		146	4.68		8445.00
**28**	35.50	63.40	0.065	0.31	0.09	660.00	120	151	174	3.54	814.25	5260.00
**29**	42.28	75.50	0.046	0.22	0.07	820.00	75	106	164	2.02	602.49	10175.00
**30**	52.86	94.40	0.060	0.29	0.07		105	88		3.03	370.75	
**31**	39.45	70.50	0.090	0.43	0.07	530.00	45	174	192	3.74	691.57	4295.00
**32**	43.74	78.10	0.063	0.30	0.04	310.00	105	153	210	3.15	379.80	2725.00

Alcohol consumed (grams), weight (kg), peak BrAC (g/210 L), peak BrAC (mg/l), peak Skyn (mg/l), peak BARE (mg/l), time-to-peak (min), and AUC. A dash (−) marks missing data for that participant and device output.

**Table 3:** Pearson’s correlations.

Device correlations	Timepoint 2 (15 min)	Timepoint 3 (30 min)	Timepoint 4 (45 min)	Timepoint 5 (60 min)	Timepoint 6 (75 min)	Timepoint 7 (90 min)	Timepoint 8 (105 min)	Timepoint 9 (120 min)	Timepoint 10 (135 min)	Timepoint 11 (150 min)	Timepoint 12 (165 min)	Timepoint 13 (180 min)	Timepoint 14 (195 min)	Timepoint 15 (210 min)	Peak TAC
**BrAC - Skyn**	***r* = 0.378,** *P* = .033, *n* = 32^*^	*r* = 0.0.94, *P* = .609, *n* = 32	*r* = 0.082, *P* = .654, *n* = 32	*r* = 0.036, *P* = .845, *n* = 32	*r* = −0.110, *P* = .549, *n* = 32	*r* = −0.196, *P* = .283, *n* = 32	*r* = −0.123, *P* = .502, *n* = 32	*r* = −0.308, *P* = .086, *n* = 32	*r* = −0.231, *P* = .203, *n* = 32	*r* = −0.103, *P* = .582, *n* = 31	*r* = −0.066, *P* = .724, *n* = 31	*r* = −0.044, *P* = .814, *n* = 31	*r* = 0.073, *P* = .698, *n* = 31	*r* = −0.153, *P* = .411, *n* = 31	*r* = −0.381, *P* = .035, *n* = 31^*^
**BrAC - BARE**	*r* = 0.196, *P* = .290, *n* = 31	*r* = 0.002, *P* = .992, *n* = 31	*r* = 0.074, *P* = .692, *n* = 31	*r* = 0.058, *P* = .758, *n* = 31	*r* = −0.078, *P* = .676, *n* = 31	*r* = 0.004, *P* = .984, *n* = 31	*r* = −0.174, *P* = .350, *n* = 31	*r* = −0.234, *P* = .205, *n* = 31	*r* = −0.244, *P* = .186, *n* = 31	*r* = −0.182, *P* = .336, *n* = 30	*r* = −0.269, *P* = .150, *n* = 30	*r* = −0.208, *P* = .270, *n* = 30	*r* = −0.173, *P* = .361, *n* = 30	*r* = −0.097, *P* = .609, *n* = 30	*r* = −0.113, *P* = .544, *n* = 31
**Skyn - BARE**	*r* = 0.038, *P* = .841, *n* = 31	*r* = 0.137, *P* = .461, *n* = 31	*r* = 0.300, *P* = .101, *n* = 31	***r* = 0.437,** ***P* = .014, *n* = 31** ^^*^^	***r* = 0.385,** ***P* = .032, *n* = 31** ^^*^^	*r* = 0.286, *P* = .119, *n* = 31	*r* = 0.273, *P* = .137, *n* = 31	*r* = 0.347, *P* = .056, *n* = 31	***r* = 0.384,** *P* = .033, *n* = 31^*^	***r* = 0.381,** *P* = .038, *n* = 30^*^	***r* = 0.384,** *P* = .036, *n* = 30^*^	***r* = 0.389,** *P* = .034, *n* = 30^*^	*r* = 0.372, *P* = .078, *n* = 30	*r* = 0.294, *P* = .115, *n* = 30	***r* = 0.380, *P* = .038, *n* = 30** ^^*^^

AUDIT, Alcohol Use Disorder Identification Test. Significant *P* < .05.

**Table 4:** Repeated measures ANOVA.

	Shapiro–Wilks Test			Mauchly’s Test of Sphericity	Greenhouse Geisser
	BrAC	Skyn	BARE		
Results	0.953, *df* = 442, *P* < .001^*^	0.949, *df* = 442, *P* < .001^*^	0.690, *df* = 442, *P* < .001^*^	*X* ^2^(2) = 6878.795, *P* = .000^*^	*F*(1.000, 441.000) = 574.820, *P* < .001^*^

^*^Significant *P* < .05.

Shapiro–Wilks test found that these data were normally distributed. A repeated measures ANOVA was conducted. Mauchly’s Test of Sphericity indicated that the assumption of sphericity had been violated *X*^2^(2) = 5070.296, *P* = .000, and therefore, a Greenhouse Geisser correction was used. The difference between the devices was statistically significant: *F*(1.00, 330.000) = 518.675, *P* < .001. Correlations with adjusted data found that lagged data from Skyn and BARE were positively correlated (*r* = 0.113, *P* = .040, *n* = 331) but still not significantly correlated to BrAC. There was a significant correlation between AUC Skyn and AUC BARE (*r* = 0.450, *P* = .013, *n* = 30). There was no significant correlation between AUC BrAC and AUC Skyn (*r* = −0.177, *P* = .341, *n* = 31) or AUC BARE (*r* = −0.106, *P* = .570, *n* = 31).

Analysing AUC up to the peak, AUC Skyn and AUC BARE were significantly positively correlated (*r* = 0.364, *P* = .044, *n* = 31).

## Discussion

This study aimed to test the accuracy of two TAS devices, compared to a breathalyser and each other. As all three devices (breathalyser, Skyn, and BARE) aim to measure alcohol consumption, we expected values from all three devices to be correlated. We predicted that the Skyn and BARE will be correlated in terms of both peak data and time-to-peak readings. Our results found that both TASs showed reactivity to the moderate amount of alcohol consumed with the output increasing rapidly for each participant. Data measured by Skyn and BARE were significantly correlated. Peak data and the AUC for Skyn and BARE were also significantly correlated and there was no significant difference in their time-to-peak. These results suggest that these TAS are reliably measuring the same phenomena. However, neither remained positively correlated to BrAC in peak or time-to-peak. We expected to see more consistent and stronger correlations between the TAS and BrAC. This could be due to the different ways that they measure alcohol, one through the breath and the other through the skin after being metabolized. In addition, this study only measured 3.5 h and so did not capture the entire drinking event with output returning to zero.

Our results show that there were no significant correlations between BARE and BrAC. In practical terms, this may lead to caution about BARE use with researchers and clinicians if BARE is not shown to be accurate and reliable for measuring alcohol consumption. However, we found that the two TASs were well correlated and appeared to be measuring the same events, significantly correlated with peak data, time-to-peak, AUC, and many of the timepoints across the afternoon. As stated, there will be a slight difference between TAS data versus BrAC due to their differences and TAS requiring alcohol metabolism before detection. The Skyn has had far more time and testing (Fairbairn and Kang 2019; Wang et al. 2019, 2021; Fairbairn et al. 2020; Rosenberg et al. 2021; Ariss et al. 2022; Ash et al. 2022; Courtney et al. 2022; Richards et al. 2022), compared to BARE, which is still in an early prototype stage. Considering this, we would have expected BARE to have a much higher failure rate than we found and a longer time-to-peak. The BARE failure rate of 4.8% is far lower than earlier Skyn prototype studies (Fairbairn and Kang 2019; Fairbairn et al. 2020) and there was no significant difference between Skyn and BARE for peak and time-to-peak data. This is encouraging for BARE’s technology at this early phase. However, comparing BARE prototype accuracy to Skyn prototype accuracy, the Skyn still showed significant positive correlations to self-report and BrAC (Fairbairn and Kang 2019; Wang et al. 2019; Fairbairn et al. 2020) in its prototype phase.

Another factor for practical use is access to the TAS output. Skyn currently requires data download once every 72 h and access to a suitable pairing device (an up-to-date Apple iOS). If the wearer does not own an iPhone to sync it themselves, regular meetings with service or hospital staff would be required for data download, increasing resources, effort, and burden for both groups. If data are not successfully downloaded in time, then it will be written over. If the wearer owns an Apple iOS device, they can use the Skyn app and see data in near-real time. However, access to real-time data for the patient may not be wanted (by the Skyn wearer or in a research setting). In addition, owning the latest Apple products in clinical addiction populations may not be as high as among the general public (Milward et al. 2015). BARE does not require a smartphone, and near real-time viewing of BARE data is not currently possible. At present, BARE can hold data for ~7 days, so would require weekly downloads.

This is not the first study to report some disagreement or smaller correlations between TAS and BrAC, BAC or self-report. Sakai et al. (2006) found that while each laboratory participant had alcohol-positive TAC and that SCRAM could distinguish between low and high alcohol dosing, individual TAC readings were not reliably equivalent to participant BrAC measurements. Then Marques and McKnight (2009) reported poor performance in the WrisTAS, with a small true positive hit rate of detecting alcohol events at 24% and another study, using WrisTAS, found the TAS to be less reliable among adolescent females (≤20 years) (Croff et al. 2020). In the agreement of self-reported and TAC for a drinking event, TAC and eBAC were moderately correlated (*r* = 0.49–0.57). Croff et al. (2020) suggest that from their results, while TAS can provide more detailed data and accurate eBAC than self-report, it may be better for reporting the frequency of drinking events. In addition, there are details that self-report can record that TAS cannot, for example, the type of alcohol being consumed. Our study had a range of ages between 18 and 47 years and a mean age of 26 years. Our results could reflect this suggestion of TAS data being less reliable in adolescents and younger adults. However, these studies used WrisTAS or SCRAM, yet the BACtrack has previously been found to perform better than SCRAM when using a model for estimating real-time BrAC measurements (Fairbairn et al. 2020) and high correlations (Fairbairn and Kang 2019; Rosenberg et al. 2021; Wang et al. 2021).

If TAS did not provide reliable output, then this would have consequences for its use. If used in clinical treatment as a motivational tool for alcohol reduction and the TAC was inaccurate, this could diminish accurate reinforcement (Stitzer and Petry 2006), ultimately reducing motivation to reduce and achieve abstinence. Or, if TAS reported an alcohol event when they were abstinent, then the individual would not receive the positive reinforcement earned. Reinforcement interventions are effective in substance use treatment but completing them incorrectly could reduce the efficiency of behaviour change (Davis et al. 2016). Within hospitals, before specific alcohol-related operations, proof of abstinence of up to 3 months may be required. TAS could be used in combination with hospital and addiction service care as proof of abstinence. If the TAS was incorrect, then this could seriously impact their treatment. Also, if TAS underreported alcohol consumption when used with the public for general health information, they could end up consuming more than they think they are, negatively impacting their health.

A current challenge of TAS is output interpretation. These two TASs provide values as TAC μg/l (air) (Skyn) or as mg/dl (BARE) and, at the time of this study, these TAS companies provided no rules to determine a drinking event, high, moderate, or low drinking levels or direct comparison to know the number of drinks consumed or BrAC comparison from the output (Wang et al. 2019). A formula could be created, similar to SCRAM rules to calculate drinking events. SCRAM provides a determination of whether alcohol was consumed, and the criteria (peak TAC, absorption, and elimination rates) which have been successfully used in research (Barnett et al., 2011, 2014, 2017; Fairbairn and Kang 2019; Fairbairn et al. 2020) and is currently used in the criminal justice system. Gunn et al. recent publication addresses some of the same challenges we found with Skyn and details useful recommendations, including the use of software (TASMAC version 2.0) to process Skyn output (Gunn et al. 2023). However, this publication appeared after our data analysis was completed. In addition, since this study was conducted, BACtrack reported changes to the capability of providing a BAC equivalent to TAC. In time, it is likely other TAS will also provide this comparison. Skyn output being formulated by software rather than manually by a researcher would improve Skyn data collection and analysis. However, due to how TAS work (alcohol consumption, metabolism, to the appearance of alcohol in this sweat vapour), there will always be a slight delay for TAC compared to BrAC, with TAC having a longer elimination time (Karns-Wright et al. 2017; Norman et al. 2020). It also could be argued that outside of research settings currently investigating TAC versus BrAC or BAC, that TAC comparison to BrAC is not necessary. If TAS can use its output and determine its own rules for alcohol detection of events, tampering, and removal, such as SCRAM has, then it may not be necessary for there to be a comparable scale to other methods, for example, a breathalyser. Each of these tools (TAS, breathalysers, blood/urine tests, self-report) have their own scales and within use for treatment and personal aid, it could be deemed that a scale to compare all against each other is not as important. This study, and previous (Swift et al. 1992; Sakai et al. 2006; Marques and McKnight 2009; Barnett et al. 2014, 2017; Bond et al. 2014; Hill-Kapturczak et al. 2014; Karns-Wright et al. 2018; Fairbairn and Kang 2019; Rash et al. 2019; Croff et al. 2020; Fairbairn et al. 2020; Rosenberg et al. 2021; Wang et al. 2021; Ash et al. 2022), show how TAS can detect and react to alcohol consumption and show the curve of a drinking event which is the proposed purpose of their use in clinical settings. This curve of data TAS can show includes information such as the speed of intoxication and duration of intoxication and presence of alcohol through the TAC, features that are not possible with self-reported data. Therefore, even without TAC conversion to BAC or BrAC, TAS has already been shown to be predictive of alcohol consumption (Russell et al. 2022).

Theorized TAS settings of use include alcohol monitoring in the criminal justice system, clinical monitoring in alcohol treatment, monitoring consumption when driving, and monitoring alcohol consumption for healthcare reasons, including general health information. SCRAM is already being used in the USA, England, and Wales for use in the criminal justice system. No country has yet implemented them as a tool for clinical practice, to our knowledge. Our findings suggest that there is still further investigation required to do for TAS to report highly consistent and accurate data over extended periods. Yet, even if they are considered sufficiently accurate and reliable to monitor alcohol consumption, using them within our laboratory study for one afternoon we found we had other considerations, such as missing data, device removals, and ease of use, which could impact their efficiency as a clinical tool (Gunn et al. 2023). There is also limited work on their acceptability, feasibility, and adherence within clinical populations and identifying facilitators and barriers to TAS clinical use which need further exploration (Brobbin et al. 2022a; Richards et al. 2023).

While this study adds to the broader literature on testing TAS in controlled settings with healthy participants, (Dougherty et al. 2012; Hill-Kapturczak et al. 2014; Roache et al. 2015, 2019; Karns-Wright et al. 2017; Fairbairn and Kang 2019; Fairbairn et al. 2020), limited work has subsequently occurred within specialist community alcohol treatment clinical settings (Brobbin et al. 2022a). Considering clinical treatment is a posited use of TAS, it must be considered why more studies have not proceeded to test accuracy and use in clinical settings. This could be due to a factor about the TAS itself or research-related challenges. Further research should explore TAS accuracy: (i) within the intended settings, (ii) over longer periods, and (iii) capture whole drinking events with typical drinking amounts that are larger amounts and longer time frames than those able to be ethically provided in laboratories. For example, those who consume alcohol for 24 h.

It will also be important to continue to examine the technological advancements of BARE. As mentioned, its specific clinical targeted use and features discussed make it appealing for clinical treatment, overcoming some of the burdens of other TAS and reduced size compared to SCRAM. As seen with Skyn and other TAS, technology advancements have led to improved output, failure rate and time-to-peak (Sakai et al. 2006; Fairbairn et al. 2019; Fairbairn and Kang 2019; Wang et al. 2019; Norman et al. 2020; Rosenberg et al. 2021). Alongside use in clinical treatment within alcohol services, TAS could be used in other medical contexts. Here further research is needed to consider if knowing if there has been alcohol consumed is enough or if specifically, how much has been consumed is also important and necessary. This study is the first to publish on BARE but we are aware of its limitations and that further assessment is required on all aspects of BARE use in clinical settings.

### Limitations

With ethical considerations, we gave participants a restricted amount of alcohol that they reported regularly consuming, and we did not exceed this amount. Outside of the laboratory individuals might be drinking larger quantities of alcohol. So, accuracy at higher quantities will need to be investigated ethically, in the future. It would also be useful to investigate the accuracy of small doses of alcohol. Due to study restrictions and the amount of alcohol participants consumed the study ended 3.5 h after alcohol consumption rather than requiring participants to stay until their BrAC reached zero. Participants were able to leave after the 3.5 h and if their BrAC was under 0.25 mg/l (a safe limit). For most when leaving, their BrAC was below 0.20 mg/l. So, although this study captured most of the TAC curve, it was not complete. This should be considered regarding the analysis, in particular when calculating the AUC.

Previous accuracy studies on Skyn have found significant correlations between Skyn and self-report (Rosenberg et al. 2021) or BrAC (Fairbairn and Kang 2019). Fairbairn and Kang (2019) used a similar design as this study, yet they found strong correlations between Skyn and BrAC peak and AUC correlations, while we did not. Differences between this study and theirs include: they used a larger sample size, had a control sample, participants were required to abstain from food for longer beforehand, were provided with a set meal based on weight and wore the devices for longer prealcohol consumption. These differences could have had an impact on TAS output due to variability in individual differences affecting alcohol metabolism. If factors such as how recently someone has eaten, or the quantity eaten significantly affect TAC then this could have implications on the TAS accuracy and practical issues of using TAS data in clinical treatment (van Egmond et al. 2020). Fairbairn and Kang (2019) also had participants stay between 3 and 4 h postalcohol, as we did, meaning their participants did not stay until BrAC returned to zero and therefore an incomplete AUC.

To note, we used the latest generation Skyn device and the BARE prototype.

## Supplementary Material

Supp_info_agad068

## Data Availability

The data that support the findings of this study are available from the corresponding author, [EB], upon reasonable request.
