# Supplementary material for: Accuracy of transdermal alcohol monitoring devices in a laboratory setting
Source: Alcohol Alcohol. 2023 Oct 23;59(1):agad068. doi: 10.1093/alcalc/agad068 (PMC12461868; doi:10.1093/alcalc/agad068)
Supplement: Supp_info_agad068 [file supp_info_agad068.docx]

**1. TAS devices: Skyn and BARE.**

|  | **Skyn by BACtrack** | **BARE by Smart Start** |
| --- | --- | --- |
| **Date available from** | Available for research use globally since 2017. | Current prototype. Announced November 2018. |
| **Company based** | United States. | United States. |
| **Target audience and purpose** | Commercial use, currently available at $199/month for research purposes only. CE marked. | Clinical research. |
| **How they work** | Fuel-cell alcohol sensor. | Transense^TM^ (Smart Start transdermal alcohol sensor). |
| **Measure TAC** | Measures TAC at 20 seconds, 1 minute or 5-minute intervals. The device can be paired to an app on a nearby Apple iOS based device. For data download, the Apple device, the data can be downloaded and viewed on an online server. | Reports TAC at 2-minute intervals, averaging 20-second data collected. Stored on the device until connected via cable to a Laptop to be downloaded to a Excel Spreadsheet. |
| **Wear** | Wrist, removable by user. Magnetic metal strap. | Wrist, removable by user. Traditional buckle watch strap. |
| **Shower/**  **bathing** | Must be removed for showering. | Must be removed for showering. |
| **Data storage** | 72 hours (approximately). | 7 days (approximately). |
| **Battery** | 10 days. | 7 days. |
| **Other information** | Also records skin temperature (Celsius). | Also records heart rate, proximity (to monitor sensor fit to skin), skin temperature (Celsius), accelerometer (movement, step count, tap sense).  The seal and absorbent pad should be removed and replaced with a new one between users. |
| **Image of device** | 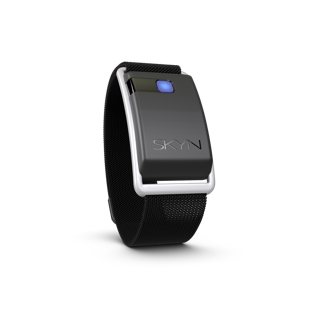<https://skyn.bactrack.com> | 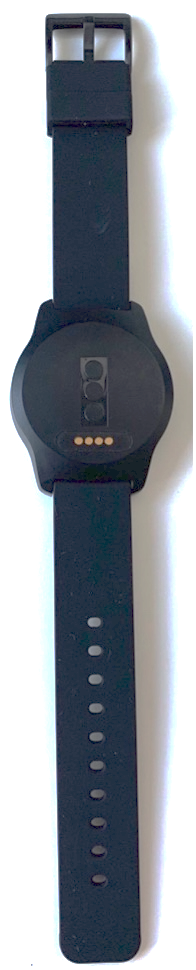 |
